# Ventral Tegmental Area Inactivation Suppresses the Expression of CA1 Long Term Potentiation in Anesthetized Rat

**DOI:** 10.1371/journal.pone.0058844

**Published:** 2013-03-11

**Authors:** Elham Ghanbarian, Fereshteh Motamedi

**Affiliations:** 1 School of Cognitive Sciences (SCS), Institute for Research in Fundamental Sciences (IPM), Tehran, Iran; 2 Neuroscience Research Center and Department of Physiology, Faculty of Medicine, Shahid Beheshti University of Medical Sciences, Tehran, Iran; Roma Tre University, Italy

## Abstract

The hippocampus receives dopaminergic projections from the ventral tegmental area (VTA). Modulatory effect of dopamine on hippocampal long term potentiation (LTP) has been studied before, but there are conflicting results and some limitations in previous reports. Most of these studies show a significant effect of dopamine on the late phase of LTP in CA1 area of the hippocampus, while few reports show an effect on the early phase. Moreover, they generally manipulated dopamine receptors in the hippocampus and there are few studies investigating influence of the VTA neural activity on hippocampal LTP in the intact brain. Besides, VTA neurons contain other neurotransmitters such as glutamate and GABA that may modify the net effect of dopamine. In this study we examined the effect of VTA reversible inactivation on the induction and maintenance of early LTP in the CA1 area of anesthetized rats, and also on different phases of learning of a passive avoidance (PA) task. We found that inactivation of the VTA by lidocaine had no effect on CA1 LTP induction and paired-pulse facilitation, but its inactivation immediately after tetanic stimulation transiently suppressed the expression of LTP. Blockade of the VTA 20 min after tetanic stimulation had no effect on the magnitude of LTP. Moreover, VTA inactivation immediately after training impaired memory in the passive avoidance task, while its blockade before or 20 min after training produced no memory deficit. It can be concluded that VTA activity has no effect on CA1 LTP induction and acquisition of PA task, but involves in the expression of LTP and PA memory consolidation.

## Introduction

Hippocampal long term potentiation (LTP), the main experimental model for the synaptic changes underlying learning and memory [1], is under the influence of ascending neuromodulatory systems. Studies of LTP, mostly in CA1, show that persistence of LTP depends not only on the two factors of the Hebbian condition (presynaptic input and postsynaptic activity), but also on the action of neurotransmitter dopamine [2]. Dopamine D1/D5 receptor agonists facilitate induction of LTP in the CA1 region of hippocampal slices [3], anesthetized [4], and freely moving rats [5], while genetic inactivation [6], [7] or pharmacological blockade of D1/D5 receptors impairs CA1 LTP [3], [8].

Dopaminergic projections from the ventral tegmental area (VTA), the area which mediates rewarding and motivated behaviors [2], differentially innervate subregions of the hippocampus so that CA1 area receives more dopamine input than CA3 or dentate gyrus [9]. The function of these projections is largely unknown; however, it has been proposed that VTA and hippocampus form a functional loop to control the entry of behaviorally significant information into long term memory [10]. This hypothesis is further supported by human fMRI studies showing there is a functional connectivity between VTA and hippocampus [11], and in reward-motivated learning, VTA activity precedes and potentiates hippocampal memory formation [2], [12], [13]. Moreover, rodent behavioral studies indicate VTA activity is necessary for spatial learning tasks, which are assumed primarily to depend on the function of the hippocampus. For example, disruption of the VTA impairs CA1 place field stability [14] and Morris water maze task [15], and D1/D5 receptor inactivation produces deficits in different spatial and associative learning tasks [6], [7], [16].

In addition to dopamine, VTA contains GABA, glutamate and a group of dopamine neurons that co-release glutamate or GABA [17]–[19] which along with dopamine neurons project to the limbic targets [17], [18] and may alter the net effect of dopamine. A number of studies using labeling techniques have shown that small percentage (15–18%) of VTA-hippocampal projections is dopaminergic but the identity of the remaining fibers has not been clearly explained [9], [20]. So far, the effect of VTA neural activity, as the main source of hippocampal dopamine [9], on LTP in the hippocampus of an intact brain has been rarely examined. One study has shown that nicotine induced LTP in dentate gyrus is mediated by activity of the VTA [21], and a very recent study reported dopaminergic lesion of VTA/SNc impairs CA1 LTP in hippocampal slices [22]. Since VTA and hippocampus are both proposed to be involved in novelty detection [10] and pathological behaviors such as drug addiction [21], [23], a better understanding of their interaction is a topic of interest and may have considerable practical implications. Here we examined the effect of VTA reversible inactivation on CA1 LTP in anesthetized rat as well as learning of passive avoidance (PA), a hippocampal dependent task [24], [25]. Reversible inactivation method by using a local anesthetic drug with a quick onset, short time effect and rapid reversibility, provides us the opportunity to observe the hippocampal synaptic response before, during, and after transient blockade of VTA [26], and the effect of a short time inactivation of the VTA on different phases of PA learning.

## Materials and Methods

### Ethics Statement

All experimental procedures complied with the guidelines of the National Institutes of Health and the Iranian Society for Physiology. The study protocol was approved by the ethics committee of Neuroscience Research Center, Shahid Beheshti University of Medical Sciences under permit number 08-06-85174411.

### Animals

Adult male Wister rats weighing 250–300 g were obtained from breeding colony of the Neuroscience Research Center, Shahid Beheshti University of Medical Sciences, Tehran, Iran. In each cage, four rats were housed and maintained at a constant temperature of 22±1°C with a 12:12-h light/dark cycle beginning with lights on at 7:00 A.M. Food and water were available ad libitum in the home cages. All experiments were carried out between 9:00 A.M. and 6:00 P.M.

### Surgical Procedures

Animals were deprived of food and water for 12 h prior to surgery. Rats were anesthetized with urethane (1.5 g/kg, i.p.) and placed in a stereotaxic frame (Stoelting, USA) for surgery and recording. Supplementary injections of urethane (0.2–0.5 g/kg) were given when necessary to ensure full anesthesia. A heating pad was used to maintain the temperature of the animals at 36.5±0.5°C. Two small holes (1 mm diameter) were drilled in the skull at the position of left dorsal hippocampus for stimulating and recording electrodes, and one for a guide cannula at the position of the VTA. A guide cannula (12 mm, 23-gauge) was implanted unilaterally above the VTA (AP: 5.4 mm posterior to bregma, ML: 0.6 mm left to the midline, and DV: 7–7.1 mm from the skull surface based on Paxinos and Watson atlas [27]). The cannula was fixed to the skull with dental cement and the exposed cortex was kept moist by the application of warm mineral oil. A bipolar stimulating electrode, a pair of twisted stainless steel Teflon-coated wires (125 μm inner diameter/150 μm external diameter, Advent Co., UK) with tips horizontally separated 500 μm apart was positioned at the Schaffer collateral pathway (AP: 3.1 mm posterior to bregma, ML: 3.1 mm, DV: 2.9–3.6 mm from the skull surface), and a similar recording electrode was positioned at the CA1 area of the dorsal hippocampus (AP: 2.8 mm posterior to bregma, ML: 1.8 mm, DV: 3.0–3.8 mm from the skull surface). The dura was pierced through both holes, and the recording and stimulating electrodes were lowered very slowly (0.2 mm/min) through the cortex and upper layers of the hippocampus into the CA1 stratum radiatum and the Schaffer collaterals, respectively. For behavioral study, animals were anesthetized with i.p. injection of a mixture of Ketamine (100 mg/kg) and Xylazine (2.5 mg/kg). Two cannulas were implanted bilaterally into the VTA and fixed to the skull with two jewelers' screws and dental cement and then animals were placed in their home cage for a recovery period of 7 days. During surgery all efforts were made to minimize animal suffering.

### Electrophysiological Recordings

The field excitatory postsynaptic potential (fEPSP) slope was used as a measure of excitatory synaptic transmission in the CA1stratum radiatum. Single 0.2 ms monophasic square wave pulses generated by a constant current isolation unit (A365, WPI, USA) were applied to the Schaffer collaterals and evoked responses were generated in the stratum radiatum. Extracellular field potentials were amplified (×1000; differential amplifier DAM 80, WPI, USA), band pass filtered between 1 Hz and 3 kHz, digitized at 10 kHz, recorded and analyzed using a homemade software. The electrodes were lowered until the appearance of a negative deflecting fEPSP with the maximum response. To measure synaptic efficacy, the fEPSP slope was calculated as the maximum slope between the initial point of the fEPSP and the peak negative response. Preparations with maximum fEPSP amplitude <1.5 mV at the maximum stimulus current or with population spikes were rejected. The optimal placement of the electrodes was determined by using electrophysiological criteria and was routinely verified by post-mortem examination. After the final determination of electrode placement, a minimum of 2 h was allowed to ensure stabilization of signal before measurements were collected. At the beginning of each experiment, input–output curve with stimulus intensities ranging from 100 to 900 μA was generated to determine the maximum fEPSP slope, and then the intensity of test stimulus was set at a level that evoked an fEPSP slope of 40% of the maximum. Test stimulation was then applied every 5 min before and after tetanic stimulation. For each time point measured during the experiments, five records of evoked responses at the frequency of 0.033 Hz were averaged. Baseline activity was measured every 5 min for at least 2 h to ensure stable baseline. The last 30 min of the baseline recording (6 time points), immediately before drug application or tetanic stimulation, was averaged and used as control for LTP induction [28]. The high frequency stimulation (HFS) protocol for inducing LTP consisted of 10 stimulus trains of 20 square wave pulses (0.2 ms duration) at 200 Hz (5 ms inter-stimulus interval) with 2 seconds inter-train interval. The magnitude of potentiation was expressed as the percentage of increase in the fEPSP slopes at the time points after tetanic stimulation relative to the slopes averaged over the 30-min baseline period. Induction of LTP was defined as increased fEPSP slope by more than 20% for at least 2 h after the HFS train. All data points were expressed as the mean ± SEM. Paired-pulse facilitation (PPF) was induced by delivering two stimuli with a 20, 50 and 100-ms inter-pulse interval at the test stimulus intensity. PPF index was defined as the ratio of the second response slope to the first one. In each experiment, average of fEPSP slopes of 5 paired-pulses delivered at the frequency of 0.033 Hz was measured. Further analysis was performed on the averages of responses.

### Microinjection Procedures

Lidocaine (0.5 μl of 4% solution in saline), an Na^+^ channel blocker, was used to temporally inactivate the VTA. The volume of injection was determined on the basis of previous reports [29]–[30]. Injection needle (30-gauge) connected to a 5-μl Hamilton syringe by a short piece of polyethylene tubing, was inserted to the guide cannula, extending 1 mm below its tip. In electrophysiological experiments, 0.5 μl of saline or lidocaine was injected slowly over 1.5 min. The injection needle was then left in the place for an additional 1.5 min to achieve a proper diffusion of the drug from its tip. Once injection finished, needle, tube and syringe were again checked to ensure they were open during injection. The behavioral experiments were carried out between 1:00 P.M. and 6:00 P.M. Rats were given at least 7 days to recover before the start of behavioral testing and during this period were handled for 3 min every day to avoid emotional stress. In the training day 0.5 µl saline or lidocaine was injected bilaterally either before or after training slowly during 5 min (2 min for each side).

### Step-Through Passive Avoidance (PA) Task

This test was performed as described previously [31]. The apparatus consisted of an illuminated chamber (20 cm×40 cm×20 cm) made from transparent plastic that was connected by a rectangular opaque guillotine door (8 cm×8 cm) to the dark compartment of the same dimensions with black opaque walls and ceiling. The floor of both chambers was made of stainless steel rods (3 mm diameter) spaced 1 cm apart. The floor of the dark chamber could be electrified. The shock was delivered to the animal's feet via a shock generator. ***Training trials*** **:** All experimental groups were given two trials to habituate them to the apparatus. For these trials, rats were placed in the illuminated compartment of apparatus facing away from the door and 10 s later, the guillotine door was raised. After the rats entered the dark compartment, the door was closed and 30 s later animals were taken from the dark compartment into their home cage. The habituation trial was repeated after 30 min and followed after the same interval by the first acquisition trial. During the adaptation trials, latency to enter the dark compartment was measured to ensure that all animals entered the dark compartment in 60 s. In the training session, each rat was placed in the light chamber and after the animal had spontaneously entered the dark compartment, the door was closed, and an electric constant current foot shock (50 Hz square wave, 1 mA for 1.5 s) was delivered through the grid floor. The entrance latency to the dark compartment, step-through latency (STL), was recorded when the animal placed all four paws in the dark compartment. The animal was removed from the dark compartment about 30 s after receiving the shock and returned to its home cage, and the trial was repeated 2 min later. This procedure was performed until the rat stayed in the lighted compartment continuously for 120 s. Depending on the number of trials to reach this criterion, training usually lasted around 10–15 minutes. Three different experiments were done: in one experiment, drug was injected before training (injection was started 5 min before training and lasted 5 min) and in two other experiments drug was injected either immediately or 20 min after training. ***Retention test:*** The retention test was performed 24 h after PA acquisition trials in the same box without any electrical shock. The rat was placed in the illuminated chamber as in the training procedure and 10 s later, the guillotine door was raised, and STL and time in dark compartment (TDC) were measured up to 300 ms.

### Histological Procedures

**Figure 1:**
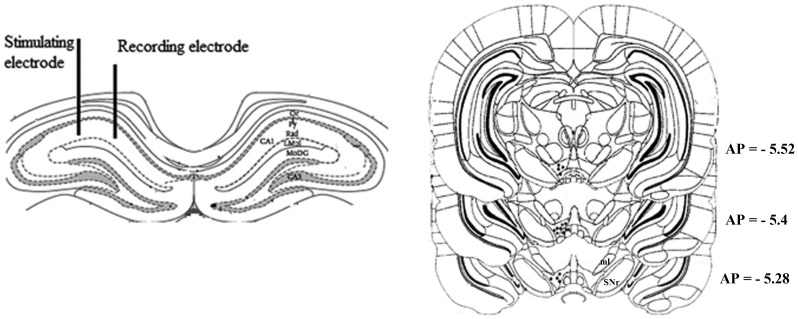
Schematic illustration of the location of electrodes and injection sites. Schematic representation of the location of electrodes in the dorsal hippocampus and the injection sites in the VTA in serial sections of the brain reconstructed from Paxinos and Watson, 2005. The numbers represent coordinates posterior to bregma.

At the end of each experiment, under diethyl ether deep anesthesia rats were decapitated by guillotine and whole brains were removed and stored in 10% formalin for at least 5 days for histological verification of the electrodes and cannula localization. Brain sections were examined using a light microscope. Brains in which an incorrect electrode or cannula localization (outside the range of 5.2–5.6 mm posterior to bregma) was found were discarded from the study. Figure 1 illustrates schematic placement of the recording and stimulating electrodes, and the injection needle tip terminating at the VTA.

### Statistical Analysis

Data were analyzed with the SPSS statistical program (SPSS, Inc., SPSS/PC+, Chicago: Illinois, USA). Induction of LTP was confirmed by comparing the average of baseline recordings before and after HFS with paired *t*-test. For analysis of difference between groups, one way or two-way analysis of variance (ANOVA) was applied. We applied analysis for 11 time points of recording; every 5 minutes for the first 30 minutes, and minutes 45, 60, 90 and 120. Statistical differences between individual time points were assessed with Student's *t*-test. For paired-pulse comparison we used paired and unpaired *t*-test. In PA learning, numbers of trials to acquisition, STL and TDC in all experiments were analyzed by Mann-Whitney *U*-test, or unpaired Student's *t*-test. Results are expressed as mean ± SEM. The significance level was set at *p*<0.05.

## Results

### Effect of VTA Inactivation on Baseline Schaffer-CA1 Synaptic Activity

**Figure 2:**
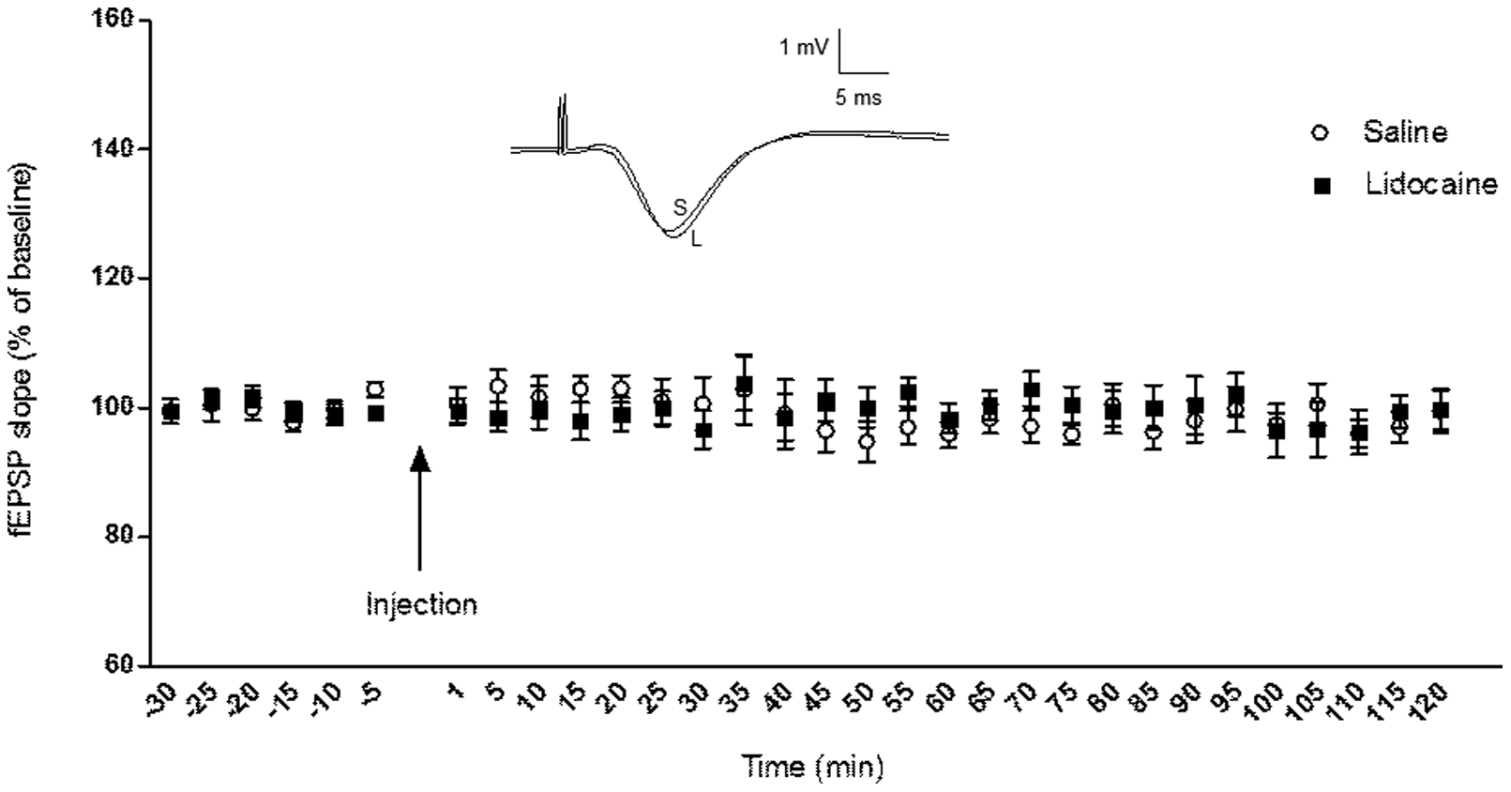
Baseline synaptic response in Schaffer–CA1 pathway is not affected by lidocaine injection into the VTA. No significant differences observed between lidocaine versus saline groups during 2 h of recording. The data are shown as mean ± SEM. Inset: Original analog traces showing evoked responses in the CA1 stratum radiatum after saline (S) and lidocaine (L) injection. The vertical scale bar corresponds to 1 mV and the horizontal to 5 ms.

No significant difference was found between baseline recording in saline (n = 6) and lidocaine (n = 7) groups (ANOVA: *F*_1, 130_ = 1.908, *p* = 0.170; Fig. 2). In addition, no significant differences between the mean fEPSP slope before and after injection were found within the lidocaine group (*t*-test: *p* = 0.860) or within the control group (*t*-test: *p* = 0.388). This finding indicates basal synaptic transmission in Schaffer-CA1 is not influenced by the VTA inactivation.

### Effect of VTA Inactivation on Paired-Pulse Facilitation in Schaffer-CA1 Synapses

**Figure 3:**
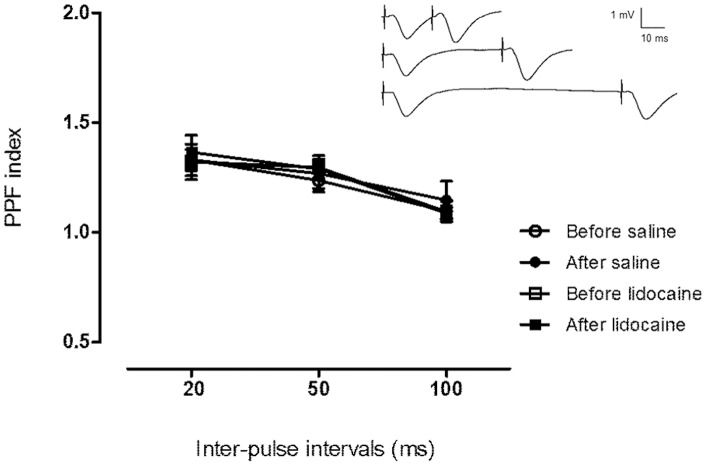
Paired-pulse facilitation of fEPSPs recorded in the CA1 stratum radiatum after stimulation of Schaffer collaterals. PPF index is defined as slopes of fEPSP2/fEPSP1. No significant differences in PPF index were observed before and after saline or lidocaine injection and between the two experimental groups at different inter-pulse intervals. Inset: Original analog traces showing paired-pulse responses in the CA1 stratum radiatum at inter-pulse intervals of 20, 50, and 100 ms. The vertical scale bar corresponds to 1 mV and the horizontal to 10 ms.

PPF was measured to evaluate the effect of VTA inactivation on the probability of transmitter release in the CA1 [32]. As shown in Fig. 3, using paired-pulse intervals of 20, 50 and 100 ms, we found no significant differences between the PPF indices of saline (n = 8) and lidocaine group (n = 7). Additionally, there was no significant difference between PPF index before and after saline or lidocaine injection into the VTA.

### Effect of VTA Inactivation on LTP Induction in the CA1 Area of the Hippocampus

**Figure 4:**
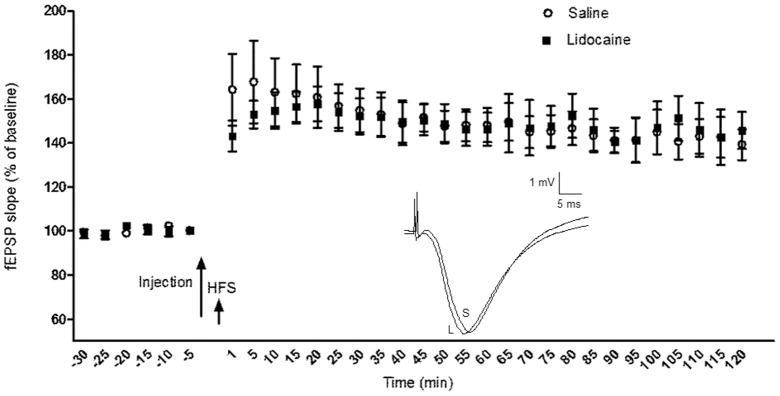
The effect of intra-VTA injection of saline or lidocaine before tetanic stimulation of the Shaffer collateral on LTP induction in the CA1 stratum radiatum. LTP was induced in both saline and lidocaine groups with no significant difference between the magnitudes of potentiation. The data are shown as mean ± SEM. Inset: Exemplar original analog traces showing LTP induction in the CA1 stratum radiatum after high frequency stimulation for saline (S) and lidocaine (L) groups. The vertical scale bar corresponds to 1 mV and the horizontal to 5 ms.

To explore the effects of VTA inputs on the induction of hippocampal LTP, we compared Schaffer-CA1 LTP in rats that received intra-VTA injection of either saline or lidocaine before tetanic stimulation. Injection procedure was started around 5 min before LTP induction to make sure it was completed before HFS. Application of 200 Hz HFS induced a significant potentiation of the fEPSP slope more than 20% of baseline levels in both groups (155.89±10.74%, paired *t*-test: *p* = 0.001, n = 8 for saline and 159.51±10.96%, paired *t*-test: *p* = 0.001, n = 9 for lidocaine; average of fEPSP slopes over 2 h of recording after HFS). No significant difference was observed in the magnitude of LTP between the two groups (ANOVA: *F*_1, 185_ = 0.318, *p* = 0.573; Fig. 4). In addition, no significant differences were found between different time points after the LTP induction within the lidocaine group (ANOVA: *F*_10, 88_ = 0.810, *p*  = 0.620) or within the control group (ANOVA: *F*_10, 77_ = 0.604, *p* = 0.806).

### Effect of VTA Inactivation on LTP Expression in the CA1 Area of the Hippocampus

**Figure 5:**
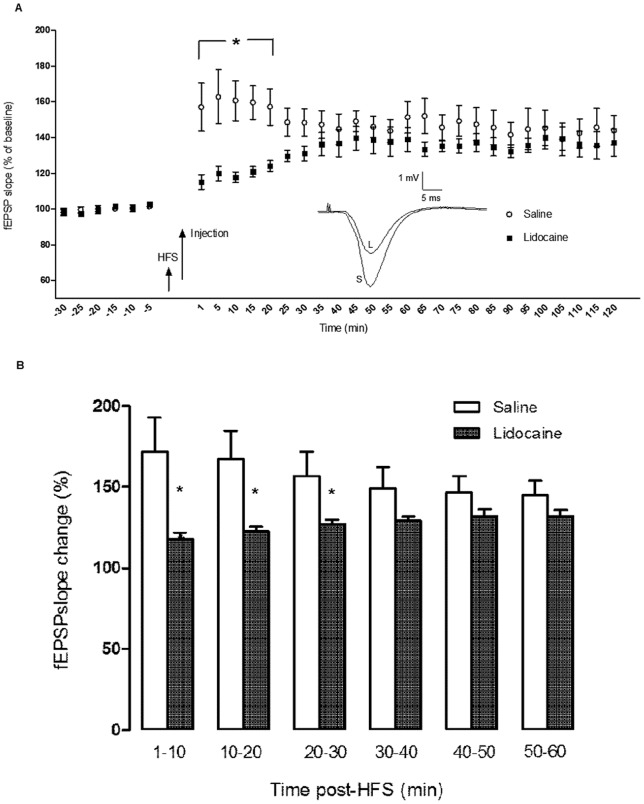
The effect of intra-VTA injection of saline or lidocaine immediately after tetanic stimulation on the expression of Schaffer-CA1 LTP. A) In both groups LTP was induced, but in the lidocaine group the magnitude of LTP was significantly lower than the control during the first 20 minutes (*p* = 0.029 for the time point of 1 min, and for the time points of 5, 10, 15 and 20 min *p* = 0.042, *p* = 0.012, *p* = 0.010, and *p* = 0.031, respectively). The data are shown as mean ± SEM. Inset: Exemplar original analog traces showing the magnitude of LTP in the saline (S) and lidocaine (L) groups for the first 20 min after HFS. The vertical scale bar corresponds to 1 mV and the horizontal to 5 ms. B) When fEPSPs were averaged in 10 min periods after the injection, difference between saline and lidocaine groups remained significant for 30 min (for 1–10 min *p* = 0.025, for 10–20 min *p* = 0.014, and for 20–30 min *p* = 0.034). Bar charts represent means ± SEM of fEPSPs.

To investigate whether inactivation of the VTA has an effect on the maintenance phase of LTP, either saline or lidocaine was injected into the VTA immediately after HFS. In both groups LTP was induced (148.70±7.56%, paired *t*-test: *p* = 0.001, n = 7 for saline and 129.89±3.19%, paired *t*-test: *p* = 0.001, n = 6 for lidocaine). However, the magnitude of LTP in the lidocaine group was significantly lower than the saline group (ANOVA: *F*_1, 141_  = 54.469, *p*<0.001). In addition, statistically significant differences were found between the different time points within the lidocaine group (ANOVA: *F*_10, 55_ = 2.659, *p* = 0.010) but not within the control group (ANOVA: *F*_10, 66_ = 0.511, *p* = 0.876). In order to explore the effect of time in more details, we compared the fEPSP slope between the two groups in each individual time point with Student's *t*-test. The maximal suppression of LTP was found immediately after the lidocaine injection, that fEPSP slope was 115.15±4.07% compared with 157.02±13.39% in the saline group (*p* = 0.029). The magnitude of LTP in the lidocaine group was significantly lower than the control group for the first 20 minutes following injection (*p* = 0.042 for the time point of 5 min, and for the time points of 10, 15 and 20 min *p* = 0.012, *p* = 0.010, and *p* = 0.031, respectively; Fig. 5A), and then gradually increased and reached the level of control LTP although it was still lower than the control level for around 1 h. Interestingly, this pattern of activity was detectable in individual response of all rats in the lidocaine group except two animals with cannula outside the VTA (misplacement controls). Moreover, the variations of responses (standard errors of means) are very small, that means VTA inactivation effectively suppresses CA1 LTP. Because the response in the lidocaine group was still lower than control group after 20 min, we averaged the magnitude of potentiation over 10 min windows and in this way the difference between two groups remained significant for 30 min (for 1–10 min *p* = 0.025, for 10–20 min *p* = 0.014, and for 20–30 min *p* = 0.034; Fig. 5B).

### Effect of VTA Inactivation 20 min Post-HFS on LTP Expression in the CA1 Area of the Hippocampus

**Figure 6:**
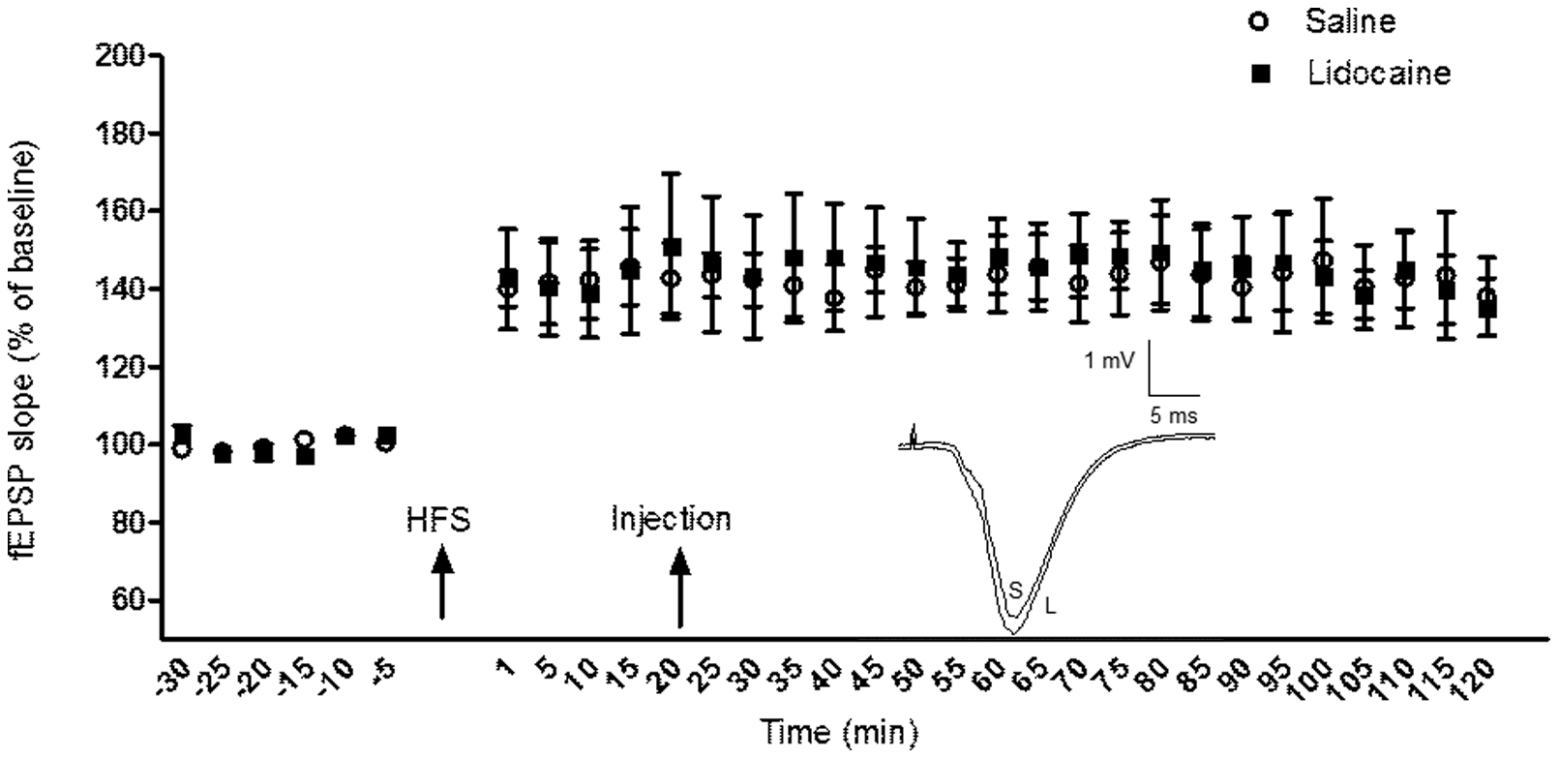
The effect of intra-VTA injection of saline or lidocaine 20 min after tetanic stimulation on the expression of Schaffer-CA1 LTP. The magnitude of LTP was not significantly different between the two groups. Different time points within each group were not significantly different as well. Inset: Exemplar original analog traces for LTP in the saline (S) and lidocaine (L) groups. The vertical scale bar corresponds to 1 mV and the horizontal to 5 ms.

In order to explore the effect of VTA inactivation on LTP expression at another time point after tetanic stimulation, in two groups of animals LTP was induced and then 20 min later either saline or lidocaine was injected. In both groups LTP was induced (141.51±7.80%, paired *t*-test: *p* = 0.003, n = 6 for saline and 144.96±10.15%, paired *t*-test: *p* = 0.011, n = 5 for lidocaine) that persisted for 2 h of recording. The magnitude of LTP was not significantly different between the two groups (ANOVA: *F*_1, 119_ = 0.143, *p* = 0.71, Fig. 6). In addition, statistically significant differences were not found between the different time points within the lidocaine group (ANOVA: *F*_10, 44_ = 0.101, *p* = 1.0) or the control group (ANOVA: *F*_10, 55_ = 0.073, *p* = 1.0).

### Effect of Pre-Training Inactivation of the VTA on Acquisition of PA

**Figure 7:**
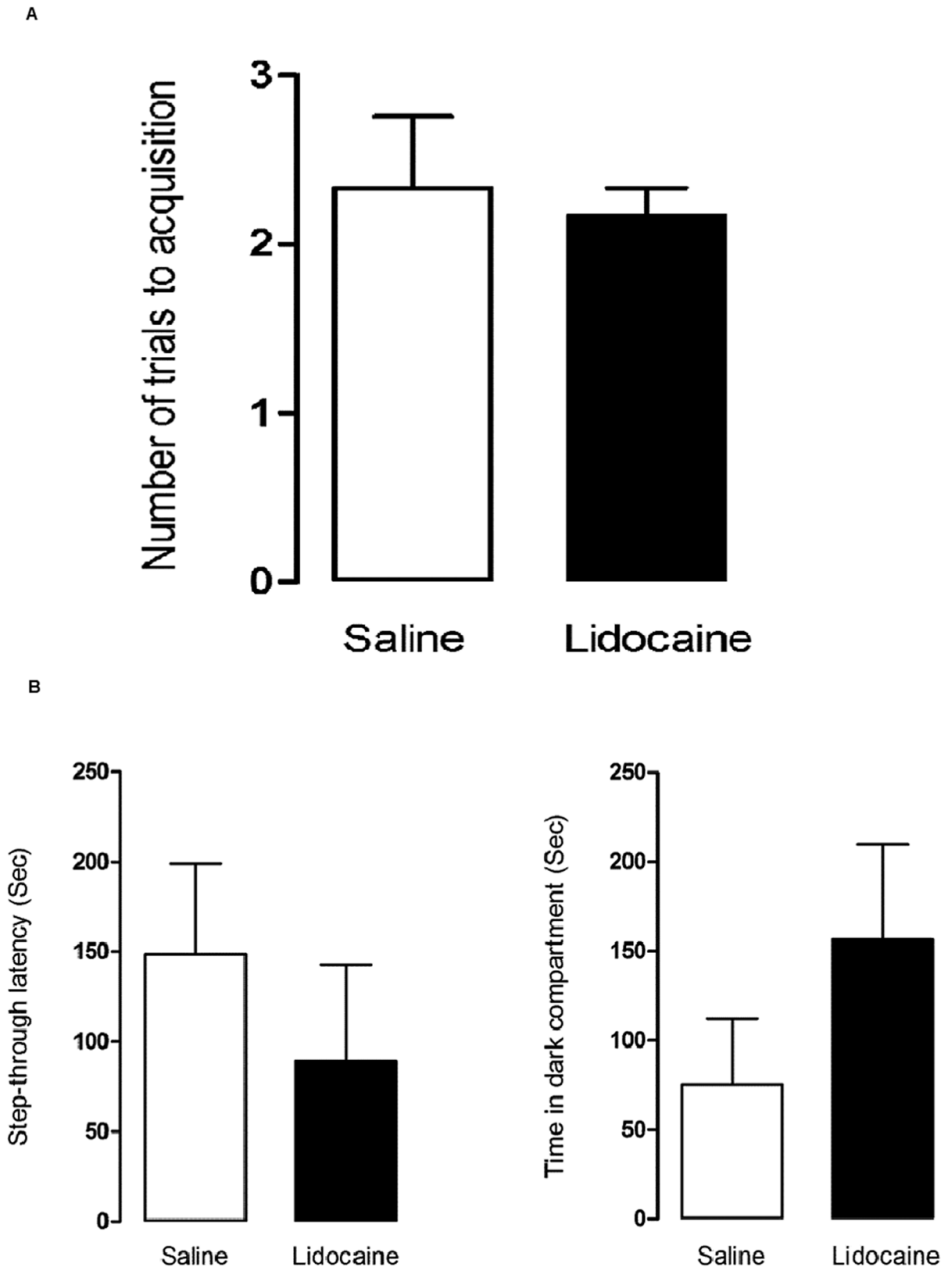
The effect of pre-training inactivation of the VTA on acquisition of PA task. A) Both saline and lidocaine groups required the same number of trials to attain criterion performance (2.33±0.42 for saline, 2.16±0.16 for lidocaine; Mann-Whitney *U*-test: *p* = 0.92), indicating that pre-training blockade of the VTA did not impair acquisition of the PA task. B) Measurement of memory 24 h after training showed STL and TDC were not statistically different between the two groups indicating pre-training inactivation of the VTA had no effect on the retention of PA, although there was a trend for lower STL and higher TDC in the lidocaine group.

To examine the effect of pre-training inactivation of the VTA, two groups of randomly selected animals received intra-VTA injection of either saline or lidocaine before training. In order to rule out the possible effect of lidocaine on the animal's motivation for entering the dark compartment as well as its locomotor activity, we first compared STL of the first and second trials of training between the two groups and found no significant difference between the groups (*p* = 0.29 & *p* = 0.36, respectively; data not shown). Then we compared the effect of pre-training intra-VTA infusion of saline (n = 6) or lidocaine (n = 6) on the number of trials necessary to reach the acquisition criterion. Both groups required the same number of trials to attain criterion performance (2.33±0.42 for the saline group, 2.16±0.16 for the lidocaine group; Mann-Whitney *U*-test: *p* = 0.92; Fig. 7A), indicating that pre-training blockade of the VTA did not impair acquisition of the PA task. Measurement of memory 24 h after training showed no significant difference between the two groups for STL (*t*-test: *p* = 0.44) and TDC (*t*-test: *p* = 0.23) indicating pre-training inactivation of the VTA had no effect on the retention of PA, although there was a trend for lower STL (89.00 ± 53.74 vs. 148.3±50.55) and higher TDC (156.5±53.28 vs. 75.33±36.96) in the lidocaine group (Fig. 7B).

### Effect of Immediate Post-Training Inactivation of the VTA on Consolidation of PA

**Figure 8:**
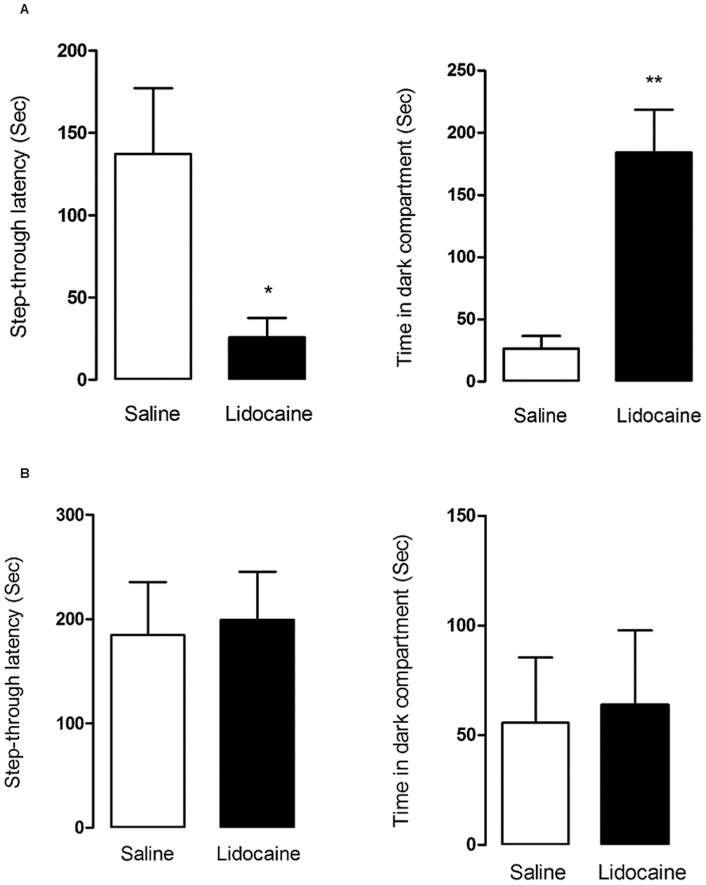
The effect of post-training inactivation of the VTA on consolidation of PA learning. A) When VTA was inactivated immediately post-training, in the retention test 24 h later the lidocaine group exhibited a significantly lower STL (*t*-test: *p* = 0.03), and a higher TDC (*t*-test: *p* = 0.005) compared to the saline group, indicating immediately post-training inactivation of the VTA impairs consolidation of PA memory. B) When VTA was inactivated 20 min after training, STL (*t*-test: *p* = 0.83) and TDC (*t*-test: *p* = 0.86) measured 24 h later were not different between saline and lidocaine groups, indicating inactivation of the VTA 20 minutes after the acquisition phase did not affect memory consolidation in PA task.

In this experiment, two groups were trained until they reached the acquisition criterion and then immediately received either saline (n = 7) or lidocaine (n = 6). The number of trials to reach the criterion was 1.85±0.20 for saline and 2.00±0.36 for lidocaine groups without a significant difference (Mann-Whitney *U*-test: *p* = 0.81). In the retention test 24 h later, the lidocaine group exhibited a significantly lower STL (25.83±11.68 vs. 137.1±39.99, *t*-test: *p* = 0.03), and a higher TDC (184.3±34.29 vs. 26.57±10.23, *t*-test: *p* = 0.005) compared to the saline group (Fig. 8A). This data indicates immediately post-training inactivation of the VTA impairs consolidation of PA memory.

### Effect of Late Post-Training Inactivation of the VTA on Consolidation of PA

Two groups of animals were trained until they acquired the criterion and 20 min later they received either saline (n = 8) or lidocaine (n = 8). The number of trials to reach the criterion was 2.00±0.26 for saline and 2.12±0.22 for lidocaine groups without a significant difference (Mann-Whitney *U*-test: *p* = 0.76). There was no significant difference between STL (*t*-test: *p* = 0.83) and TDC (*t*-test: *p* = 0.86) measured 24 h later (Fig. 8B), indicating inactivation of the VTA 20 minutes after the acquisition phase had no effect on memory consolidation in PA.

## Discussion

The main finding of this study is that pre-HFS inactivation of the VTA had no effect on CA1 LTP induction, but its post-HFS inactivation transiently suppressed the expression of LTP in anesthetized rat. VTA inactivation 20 min after tetanic stimulation had no effect on the expression of LTP. Basic synaptic response and PPF were not affected as well. In the PA task, blocking the VTA immediately after training impaired memory consolidation, while its blockade before training or 20 min after training did not produce any memory deficit. Together, these findings suggest a role for the VTA in hippocampal synaptic plasticity and memory. It has been proposed that VTA interacts with hippocampus to form long term memories [10], and VTA disruption impairs hippocampal dependent functions such as Morris water maze [15] and CA1 place field stability [14]. Our study shows a direct influence of the VTA neural activity on CA1 LTP.

Consistent with our data, previous studies found no changes in the baseline synaptic response as well as PPF in the CA1 area of anesthetized rats by inactivation [21], electrical lesion or stimulation of the VTA [33] and in D1R depleted animals [6], [7]. A recent study clearly showed there are no differences in postsynaptic currents recorded by patch clamp, extracellular field EPSP_s_ and paired-pulse ratios of field EPSP_s_ in slices from sham-operated and VTA/SNc 6-OHDA-lesioned rats [22]. Since PPF is mostly associated with the enhanced presynaptic transmitter release [32], our result likely indicates modulatory dopaminergic inputs from the VTA are not acting on the presynaptic terminals in the CA1 area. Indeed, available evidence demonstrates most D1/D5 receptors are postsynaptic particularly in the cell membrane of the CA1 pyramidal cells [34].

There is a large body of evidence for dopaminergic involvement in hippocampal LTP and learning and memory both in human [35], [36] and rodents [10]. While most of the previous studies demonstrate dopamine (DA) receptors in the CA1 area influence the late, protein synthesis-dependent phase of LTP [2], [8], fewer reports suggest that loss of DA impairs CA1 early LTP [3], [6], [7]. We found that when the VTA was inactivated immediately after tetanic stimulation, the magnitude of the early phase of LTP was decreased significantly for 20–30 min and then gradually increased to the control level. Studies on the effective time course of lidocaine show that this drug silences neural activity for 3–10 min and then complete recovery occurs within 5–15 min after the injection [37]–[39]. Therefore, it can be suggested that the magnitude of LTP was suppressed during VTA inactivation and by gradual recovery of the VTA it returned to the control level. One possible explanation for this finding could be reduction of DA in the CA1 area during VTA inactivation. Real-time measurement of dopamine transmission in the nucleus accumbens of awake rats clearly showed both electrically evoked release of DA and its naturally occurring transients were significantly attenuated following intra-VTA microinfusion of 0.5 µl lidocaine [29]. Moreover, it has been shown that 50% dopaminergic cell loss of the VTA by 6-OHDA resulted in 74% reduction of DA in the hippocampus [15]. Therefore, it can be suggested that following inactivation of the VTA dopamine concentration in the CA1 area is decreased which in turn affects LTP magnitude. In agreement with this finding, recent study by Costa *et al.* has shown unilateral 6-OHDA lesion of the SNc/VTA significantly reduced DA transmission and the magnitude of early LTP in the CA1 area recorded in hippocampal slices [22]. Also Gurden *et* *al.* has reported when electrolytic lesion of the VTA produced more than 50% DA depletion in the prefrontal cortex of freely moving rats, the magnitude of LTP in hippocampus-prefrontal synapses was decreased during the first hour following tetanus, and then slowly raised and reached the level of potentiation in the control group [40]. Short duration of LTP suppression in our experiment and its recovery to the control level after 20–30 min could be explained by increase of DA while the VTA is returning to its normal activity. On the other hand, this finding is nearly similar to what Gurden and colleagues reported, and may implicate a common compensatory mechanism in both permanent and transient lesions of the VTA in the intact brain.

On the contrary, when the VTA was inactivated before tetanic stimulation LTP was normally induced. This finding is in line with several reports indicating DA is necessary for the maintenance rather than induction of LTP [2], [8], [41]–[43]. For example, O'Carroll and Morris showed with application of DA antagonists early LTP in the CA1 area of hippocampal slices was normally induced but its maintenance was significantly impaired [41]. On the other hand, a number of studies have reported the involvement of DA in LTP induction in certain experimental conditions [4], [5], [36]. Huang and Kandel demonstrated D1/D5 antagonists depressed LTP induced by triple tetanization while had no effect on LTP induced by one train of tetanus in slices [42]. Lemon and Manahan reported when a D1/D5 receptor agonist was injected before tetanus it lowered the threshold for LTP induction and LTP was induced by a weak tetanus; however, injection of a DA antagonist before strong tetanus had no effect on induction and early phase of LTP but impaired the late phase (>3 h) in freely moving rats [28]. Therefore, the protocol used for LTP induction [44], differences between *in vitro* and *in vivo* conditions, and the method used for DA manipulation might be some possible reasons for different and sometimes paradoxical findings about the effect of DA on induction and maintenance of LTP. Moreover, in our experiments the results of pre- and post-HFS injection of lidocaine are more difficult to explain considering the fact that pre-HFS inactivation of the VTA might have influenced the initial moments of LTP expression, but have not impaired it. The underlying mechanisms of this paradox would be better understood by complementary experiments such as measurement of the VTA neural activity and DA fluctuations in the CA1 area after lidocaine injection. However, a possible mechanism can be postulated for normal expression of LTP. HFS is an artificial electrical stimulation and we have used a strong HFS protocol; therefore, the strength of HFS may have overcome the effects of (partial) blockade of the VTA. Weak, sub threshold stimulation in “learning-facilitated plasticity” [4], [45] may provide a more natural condition to measure the effects of the VTA dopaminergic signals and help elucidating this paradoxical finding.

On the other hand, the results of PA learning show that VTA inactivation did not affect acquisition but impaired memory consolidation. This finding is in line with a lesion study showing chemical lesions of the VTA did not affect acquisition of PA, while significantly decreased the STL 24 h later [46]; however, because of producing permanent lesions they could not compare the consolidation versus retention phase. Moreover, intra-hippocampal infusion of D1/D5 receptor antagonists before training did not affect the encoding of associative and spatial memories, while its infusion immediately after training impaired consolidation of these hippocampal dependent memories [16], [47]. Considering the limitations of applying interpretations of electrophysiological data to the behavioral findings, in our study we can compare the effect of VTA inactivation on acquisition and consolidation of PA with its effect on the induction and expression of LTP, respectively.

When VTA was inactivated 20 min after tetanus it did not affect LTP expression. This may suggest that pairing of inactivation with HFS in a short time period is important to see the suppression effect and once LTP has been established, blocking the VTA has no influence on its magnitude. Our behavioral findings seems to be consistent with the electrophysiological data so that inactivation of the VTA immediately after training impaired memory consolidation while its blockade 20 min after training had no effect. This data is supported by several reports that intra-hippocampal infusion of DA antagonists during or immediately after training, but not 15 min or 2 h or 3 h later impairs consolidation of spatial memory and inhibitory avoidance tasks; implying DA has no effect on previously established memories [16], [48], [49]. It has been suggested that physiological impact of dopaminergic activity is to interact with NMDA receptors at or around the time of encoding to trigger cellular consolidation processes immediately after encoding [16], [47].

Finally, VTA non-dopaminergic neurotransmitters including glutamate and GABA also might have influenced our results. Rossato *et al.* demonstrated immediate post-training activation of the VTA is essential to determine the duration of long term memory in PA task; however, this role does not involve hippocampal D1 receptors since none of the immediate manipulations of these receptors affected long term memory [30]. Bernabeu *et al.* suggested that a dopamine-independent, PKA-mediated mechanism may also be required in the immediate early phase of memory formation in the hippocampus [25]. In fact, it has been demonstrated that VTA stimulation in anesthetized rats evokes a fast, short-latency EPSP in PFC that is not mediated by dopamine, but rather by glutamate AMPA and NMDA receptors [50], [51]. These data suggest the net effect of VTA activity may not be limited to the neuromodulatory action of dopamine. Considering the small percentage of VTA-hippocampal dopaminergic fibers [9], [20], a possible influence of non-dopaminergic neurotransmitters on synaptic responses in our study could also be assumed [52].

Based on the previous studies, infusion of 0.5 μl lidocaine covers almost 70% of the VTA [29], therefore in our study lidocaine likely inactivated substantial portions of the VTA without affecting the nearby structures. However, lidocaine also inactivates fibers of passage from raphe nucleus and locus coeruleus [33], [53] that might have produced the observed LTP impairment. Previous studies showed serotonin antagonists increase the magnitude and duration of CA1 LTP [54], [55], while its agonists block it [56], [57]. On the other hand, locus coeruleus stimulation or application of noradrenergic agonists induces long term depression [58], [59] or modulate low frequency stimulation induced LTP [60], [61] at Shaffer-CA1 synapses. Therefore, inactivation of the passing fibers does not seem to produce LTP suppression in our study, although this possibility cannot be entirely ruled out without complementary experiments. Finally it should be noted that although here we have shown that VTA inactivation impairs hippocampal LTP and PA memory, further studies are required to rule out the possible role of other brain regions that receive inputs from the VTA and mediate PA response such as amygdale and nucleus accumbens [17], [62]–[64] in the observed PA memory deficit. In conclusion, VTA activity seems to be important for the expression rather than induction of hippocampal LTP, and in consolidation but not acquisition of PA learning. This study provides supporting evidence for the idea that the VTA and hippocampus form a functional loop to control the entry of newly acquired information into the storage of long term memory [10].

## References

[1] Bliss TV, Collingridge GL (1993) A synaptic model of memory: long-term potentiation in the hippocampus. Nature 361: 31–39. 8421494 10.1038/361031a0

[2] Lisman J, Grace AA, Duzel E (2011) A neoHebbian framework for episodic memory; role of dopamine-dependent late LTP. Trends Neurosci 34: 536–547. 21851992 10.1016/j.tins.2011.07.006 PMC3183413

[3] Otmakhova NA, Lisman JE (1996) D1/D5 dopamine receptor activation increases the magnitude of early long-term potentiation at CA1 hippocampal synapses. J Neurosci 16: 7478–7486. 8922403 10.1523/JNEUROSCI.16-23-07478.1996 PMC6579102

[4] Li S, Cullen WK, Anwyl R, Rowan MJ (2003) Dopamine-dependent facilitation of LTP induction in hippocampal CA1 by exposure to spatial novelty. Nat Neurosci 6: 526–531. 12704392 10.1038/nn1049

[5] Lemon N, Manahan-Vaughan D (2006) Dopamine D1/D5 receptors gate the acquisition of novel information through hippocampal long-term potentiation and long-term depression. J Neurosci 26: 7723–7729. 16855100 10.1523/JNEUROSCI.1454-06.2006 PMC6674280

[6] Granado N, Ortiz O, Suárez LM, Martín ED, Ceña V, et al (2008) D1 but not D5 dopamine receptors are critical for LTP, spatial learning, and LTP Induced arc and zif268 expression in the hippocampus. Cereb Cortex 18: 1–12. 17395606 10.1093/cercor/bhm026

[7] Ortiz O, Delgado-García JM, Espadas I, Bahí A, Trullas R, et al (2010) Associative learning and CA3-CA1 synaptic plasticity are impaired in D1R null, Drd1a−/− mice and in hippocampal siRNA silenced Drd1a mice. J Neurosci 30: 12288–12300. 20844125 10.1523/JNEUROSCI.2655-10.2010 PMC6633447

[8] Frey U, Schroeder H, Matthies H (1990) Dopaminergic antagonists prevent long-term maintenance of posttetanic LTP in the CA1 region of rat hippocampal slices. Brain Res 522: 69–75. 1977494 10.1016/0006-8993(90)91578-5

[9] Gasbarri A, Packard MG, Campana E, Pacitti C (1994) Anterograde and retrograde tracing of projections from the ventral tegmental area to the hippocampal formation in the rat. Brain Res Bull 33: 445–452. 8124582 10.1016/0361-9230(94)90288-7

[10] Lisman JE, Grace AA (2005) The hippocampal-VTA loop: controlling the entry of information into long-term memory. Neuron 46: 703–713. 15924857 10.1016/j.neuron.2005.05.002

[11] I K, D S (2012) Intrinsic connectivity between the hippocampus, nucleus accumbens, and ventral tegmental area in humans. Hippocampus (In press) doi: 10.1002/hipo.22077. 10.1002/hipo.22077 PMC4118056 23129267

[12] Wittmann BC, Schott BH, Guderian S, Frey JU, Heinze HJ, et al (2005) Reward-related fMRI activation of dopaminergic midbrain is associated with enhanced hippocampus-dependent long-term memory formation. Neuron 45: 459–467. 15694331 10.1016/j.neuron.2005.01.010

[13] Adcock RA, Thangavel A, Whitfield-Gabrieli S, Knutson B, Gabrieli JDE (2006) Reward-motivated learning: mesolimbic activation precedes memory formation. Neuron 50: 507–517. 16675403 10.1016/j.neuron.2006.03.036

[14] Martig AK, Mizumori SJ (2011) Ventral tegmental area disruption selectively affects CA1/CA2 but not CA3 place fields during a differential reward working memory task. Hippocampus 21: 172–184. 20082295 10.1002/hipo.20734 PMC2988981

[15] Wisman LA, Sahin G, Maingay M, Leanza G, Kirik D (2008) Functional convergence of dopaminergic and cholinergic input is critical for hippocampus-dependent working memory. J Neurosci 28: 7797–7807. 18667612 10.1523/JNEUROSCI.1885-08.2008 PMC6670368

[16] O'Carroll CM, Martin SJ, Sandin J, Frenguelli B, Morris RG (2006) Dopaminergic modulation of the persistence of one-trial hippocampus-dependent memory. Learn Mem 13: 760–769. 17142305 10.1101/lm.321006 PMC1783630

[17] Fields HL, Hjelmstad GO, Margolis EB, Nicola SM (2007) Ventral tegmental area neurons in learned appetitive behavior and positive reinforcement. Ann Rev Neurosci 30: 289–316. 17376009 10.1146/annurev.neuro.30.051606.094341

[18] Yamaguchi T, Wang HL, Li X, Ng TH, Morales M (2011) Mesocorticolimbic glutamatergic pathway. J Neurosci 31: 8476–8490. 21653852 10.1523/JNEUROSCI.1598-11.2011 PMC6623324

[19] Tritsch NX, Ding JB, Sabatini BL (2012) Dopaminergic neurons inhibit striatal output through non-canonical release of GABA. Nature 490: 262–266. 23034651 10.1038/nature11466 PMC3944587

[20] Swanson LW (1982) The projections of the ventral tegmental area and adjacent regions: a combined fluorescent retrograde tracer and immunofluorescence study in the rat. Brain Res Bull 9: 321–353. 6816390 10.1016/0361-9230(82)90145-9

[21] Tang J, Dani JA (2009) Dopamine enables in vivo synaptic plasticity associated with the addictive drug nicotine. Neuron 63: 673–682. 19755109 10.1016/j.neuron.2009.07.025 PMC2746116

[22] Costa C, Sgobio C, Siliquini S, Tozzi A, Tantucci M, et al (2012) Mechanisms underlying the impairment of hippocampal long-term potentiation and memory in experimental Parkinson's disease. Brain 135: 1884–1899. 22561640 10.1093/brain/aws101

[23] Everitt BJ, Robbins TW (2005) Neural systems of reinforcement for drug addiction: from actions to habits to compulsion. Nat Neurosci 8: 1481–1489. 16251991 10.1038/nn1579

[24] Lorenzini CA, Baldi E, Bucherelli C, Sacchetti B, Tassoni G (1996) Role of dorsal hippocampus in acquisition, consolidation and retrieval of rat's passive avoidance response: a tetrodotoxin functional inactivation study. Brain Res 730: 32–39. 8883885 10.1016/0006-8993(96)00427-1

[25] Bernabeu R, Bevilaqua L, Ardenghi P, Bromberg E, Schmitz P, et al (1997) Involvement of hippocampal cAMP/cAMP-dependent protein kinase signaling pathways in a late memory consolidation phase of aversively motivated learning in rats. Proc Natl Acad Sci U S A 94: 7041–7046. 9192688 10.1073/pnas.94.13.7041 PMC21281

[26] Lashgari R, Khakpour-Taleghani B, Motamedi F, Shahidi S (2008) Effects of reversible inactivation of locus coeruleus on long-term potentiation in perforant path-DG synapses in rats. Neurobiol Learn Mem 90: 309–316. 18577458 10.1016/j.nlm.2008.05.012

[27] Paxinos G, Watson C (2005) The rat brain in stereotaxic coordinates (5th ed.). San Diego: Elsevier Academic Press.

[28] Lemon N, Manahan-Vaughan D (2006) Dopamine D1/D5 receptors gate the acquisition of novel information through hippocampal long-term potentiation and long-term depression. J Neurosci 26: 7723–7729. 16855100 10.1523/JNEUROSCI.1454-06.2006 PMC6674280

[29] Sombers LA, Beyene M, Carelli RM, Wightman RM (2009) Synaptic overflow of dopamine in the nucleus accumbens arises from neuronal activity in the ventral tegmental area. J Neurosci 29: 1735–1742. 19211880 10.1523/JNEUROSCI.5562-08.2009 PMC2673986

[30] Rossato JI, Bevilaqua LR, Izquierdo I, Medina JH, Cammarota M (2009) Dopamine controls persistence of long-term memory storage. Science 325: 1017–1020. 19696353 10.1126/science.1172545

[31] Davoodi FG, Motamedi F, Akbari E, Ghanbarian E, Jila B (2011) Effect of reversible inactivation of reuniens nucleus on memory processing in passive avoidance task. Behav Brain Res 221: 1–6. 21354215 10.1016/j.bbr.2011.02.020

[32] Zucker RS (1989) Short-term synaptic plasticity. Annu Rev Neurosci 12: 13–31. 2648947 10.1146/annurev.ne.12.030189.000305

[33] Spencer PM, Wheal HV (1990) Synaptic inhibition in the rat hippocampus in vivo following stimulation of the substantia nigra and ventral tegmentum. J Physiol 423: 77–90. 1974925 10.1113/jphysiol.1990.sp018012 PMC1189747

[34] Huang Q, Zhou D, Chase K, Gusella JF, Aronin N, et al (1992) Immunohistochemical localization of the D1 dopamine receptor in rat brain reveals its axonal transport, pre- and postsynaptic localization, and prevalence in the basal ganglia, limbic system, and thalamic reticular nucleus. Proc Natl Acad Sci U S A 89: 11988–11992. 1281547 10.1073/pnas.89.24.11988 PMC50683

[35] Chowdhury R, Guitart-Masip M, Bunzeck N, Dolan RJ, Düzel E (2012) Dopamine modulates episodic memory persistence in old age. J Neurosci 32: 14193–14204. 23055489 10.1523/JNEUROSCI.1278-12.2012 PMC3734374

[36] Hamilton TJ, Wheatley BM, Sinclair DB, Bachmann M, Larkum ME, et al (2010) Dopamine modulates synaptic plasticity in dendrites of rat and human dentate granule cells. Proc Natl Acad Sci U S A 107: 18185–18190. 20921404 10.1073/pnas.1011558107 PMC2964233

[37] Malpeli JG, Schiller PH (1979) A method of reversible inactivation of small regions of brain tissue. J Neurosci Methods 1: 143–151. 120911 10.1016/0165-0270(79)90011-6

[38] Malpeli JG (1999) Reversible inactivation of subcortical sites by drug injection. J Neurosci Methods 86: 119–128. 10065981 10.1016/s0165-0270(98)00161-7

[39] Boeijinga PH, Mulder AB, Pennartz CM, Manshanden I, Lopes da Silva FH (1993) Responses of the nucleus accumbens following fornix/fimbria stimulation in the rat. Identification and long-term potentiation of mono- and polysynaptic pathways. Neuroscience 53: 1049–1058. 8389427 10.1016/0306-4522(93)90488-2

[40] Gurden H, Tassin JP, Jay TM (1999) Integrity of the mesocortical dopaminergic system is necessary for complete expression of in vivo hippocampal-prefrontal cortex long-term potentiation. Neuroscience 94: 1019–1027. 10625044 10.1016/s0306-4522(99)00395-4

[41] O'Carroll CM, Morris RG (2004) Heterosynaptic co-activation of glutamatergic and dopaminergic afferents is required to induce persistent long-term potentiation. Neuropharmacology 47: 324–332. 10.1016/j.neuropharm.2004.04.005 15275821

[42] Huang YY, Kandel ER (1995) D1/D5 receptor agonists induce a protein synthesis dependent late potentiation in the CA1 region of the hippocampus. Proc Natl Acad Sci U S A 92: 2446–2450. 7708662 10.1073/pnas.92.7.2446 PMC42234

[43] Navakkode S, Sajikumar S, Frey JU (2007) Synergistic requirements for the induction of dopaminergic D1/D5-receptor-mediated LTP in hippocampal slices of rat CA1 in vitro. Neuropharmacology 52: 1547–1554. 17433377 10.1016/j.neuropharm.2007.02.010

[44] Buschler A, Goh JJ, Manahan-Vaughan D (2012) Frequency dependency of NMDA receptor-dependent synaptic plasticity in the hippocampal CA1 region of freely behaving mice. Hippocampus 22: 2238–2248. 22707377 10.1002/hipo.22041

[45] Hansen N, Manahan-Vaughan D (2012) Dopamine D1/D5 Receptors Mediate Informational Saliency that Promotes Persistent Hippocampal Long-Term Plasticity. Cereb Cortex (In press). 10.1093/cercor/bhs362 PMC3948488 23183712

[46] Hefco V, Yamada K, Hefco A, Hritcu L, Tiron A, et al (2003) Role of the mesotelencephalic dopamine system in learning and memory processes in the rat. Eur J Pharmacol 475: 55–60. 12954359 10.1016/s0014-2999(03)02115-0

[47] Bethus I, Tse D, Morris RG (2010) Dopamine and memory: modulation of the persistence of memory for novel hippocampal NMDA receptor-dependent paired associates. J Neurosci 30: 1610–1618. 20130171 10.1523/JNEUROSCI.2721-09.2010 PMC6633999

[48] Castellano C, Cestari V, Cabib S, Puglisi-Allegra S (1991) Post-training dopamine receptor agonists and antagonists affect memory storage in mice irrespective of their selectivity for D1 or D2 receptors. Behav Neural Biol 56: 283–291. 1684703 10.1016/0163-1047(91)90439-w

[49] da Silva WC, Köhler CC, Radiske A, Cammarota M (2012) D1/D5 dopamine receptors modulate spatial memory formation. Neurobiol Learn Mem 97: 271–275. 22266268 10.1016/j.nlm.2012.01.005

[50] Stuber GD, Hnasko TS, Britt JP, Edwards RH, Bonci A (2010) Dopaminergic terminals in the nucleus accumbens but not the dorsal striatum corelease glutamate. J Neurosci 30: 8229–8233. 20554874 10.1523/JNEUROSCI.1754-10.2010 PMC2918390

[51] Tecuapetla F, Patel JC, Xenias H, English D, Tadros I, et al (2010) Glutamatergic signaling by mesolimbic dopamine neurons in the nucleus accumbens. J Neurosci 30: 7105–7110. 20484653 10.1523/JNEUROSCI.0265-10.2010 PMC3842465

[52] Lapish CC, Seamans JK, Chandler LJ (2006) Glutamate-dopamine cotransmission and reward processing in addiction. Alcohol Clin Exp Res 30: 1451–1465. 16930207 10.1111/j.1530-0277.2006.00176.x

[53] Oades RD, Halliday GM (1987) Ventral tegmental (A10) system: neurobiology. 1. Anatomy and connectivity. Brain Res 434: 117–165. 3107759 10.1016/0165-0173(87)90011-7

[54] Stäubli U, Xu FB (1995) Effects of 5-HT3 receptor antagonism on hippocampal theta rhythm, memory, and LTP induction in the freely moving rat. J Neurosci 15: 2445–2452. 7891179 10.1523/JNEUROSCI.15-03-02445.1995 PMC6578162

[55] Wang RY, Arvanov VL (1998) M100907, a highly selective 5-HT2A receptor antagonist and a potential atypical antipsychotic drug, facilitates induction of long-term potentiation in area CA1 of the rat hippocampal slice. Brain Res 779: 309–313. 9473706 10.1016/s0006-8993(97)01174-8

[56] Corradetti R, Ballerini L, Pugliese AM, Pepeu G (1992) Serotonin blocks the long-term potentiation induced by primed burst stimulation in the CA1 region of rat hippocampal slices. Neuroscience 46: 511–518. 1545909 10.1016/0306-4522(92)90140-w

[57] Shakesby AC, Anwyl R, Rowan MJ (2002) Overcoming the effects of stress on synaptic plasticity in the intact hippocampus: rapid actions of serotonergic and antidepressant agents. J Neurosci 22: 3638–3644. 11978839 10.1523/JNEUROSCI.22-09-03638.2002 PMC6758347

[58] Lemon N, Aydin-Abidin S, Funke K, Manahan-Vaughan D (2009) Locus coeruleus activation facilitates memory encoding and induces hippocampal LTD that depends on beta-adrenergic receptor activation. Cereb Cortex 19: 2827–2837. 19435710 10.1093/cercor/bhp065 PMC2774396

[59] Scheiderer CL, Dobrunz LE, McMahon LL (2004) Novel form of long-term synaptic depression in rat hippocampus induced by activation of alpha 1 adrenergic receptors. J Neurophysiol 91: 1071–1077. 14573563 10.1152/jn.00420.2003

[60] Katsuki H, Izumi Y, Zorumski CF (1997) Noradrenergic regulation of synaptic plasticity in the hippocampal CA1 region. J Neurophysiol 77: 3013–3020. 9212253 10.1152/jn.1997.77.6.3013

[61] Thomas MJ, Moody TD, Makhinson M, O'Dell TJ (1996) Activity-dependent beta-adrenergic modulation of low frequency stimulation induced LTP in the hippocampal CA1 region. Neuron 17: 475–482. 8816710 10.1016/s0896-6273(00)80179-8

[62] Oleson EB, Gentry RN, Chioma VC, Cheer JF (2012) Subsecond dopamine release in the nucleus accumbens predicts conditioned punishment and its successful avoidance. J Neurosci 32: 14804–14808. 23077064 10.1523/JNEUROSCI.3087-12.2012 PMC3498047

[63] Levita L, Dalley JW, Robbins TW (2002) Nucleus accumbens dopamine and learned fear revisited: a review and some new findings. Behav Brain Res 137: 115–127. 12445718 10.1016/s0166-4328(02)00287-5

[64] Davis M (1992) The role of the amygdala in fear and anxiety. Annu Rev Neurosci 15: 353–375. 1575447 10.1146/annurev.ne.15.030192.002033

